# Neuroleptic malignant syndrome with haloperidol and quetiapine treatment in a schizophrenia patient: A case report and 1 year follow up

**DOI:** 10.1192/j.eurpsy.2023.2133

**Published:** 2023-07-19

**Authors:** F. Alioglu Karayilan, B. Yildiz, I. Ak

**Affiliations:** 1 Psychiatry, Private Praxis; 2 Psychiatry, Karabuk University, Karabuk, Türkiye

## Abstract

**Introduction:**

Neuroleptic malignant syndrome is a rare but also life-threatening adverse reaction associated with mostly antipsychotic use. It is mostly related with the administration of D2 dopamine receptor-blocking antipsychotics or sudden discontinuation of antiparkinsonian drugs.

**Objectives:**

In this article, we present a 55-year-old male patient diagnosed with schizophrenia who was admitted to the emergency department with acute onset confusion and fever. He had been on haloperidol and quetiapine treatment for several years.

**Methods:**

His Creatine kinase (CK) levels were elevated (2928 - 11462 U/l within 1 day. U/l). She was admitted to the intensive care unit with an initial diagnosis of NMS. The patient’s all antipsychotic treatment was discontinued.

**Results:**

At a follow-up examination after 6 days, myoglobin, CK, and leukocyte values were noted to start decreasing. The rigidity and altered mental status improved after 6 days. After 9 days he was discharged. He was admitted to the psychiatry outpatient clinic. He was suffering from auditory hallucinations therefore the antipsychotic treatment has been started and it was planned to reach the target dose of 10mg olanzapine PO by increasing the weekly dose to 2.5 mg. However, due to the increase in the patient’s auditory hallucinations, the treatment was rearranged and the target dose of 15mg was reached. After that, the patient’s positive symptoms regressed significantly. The patient has been on 15 mg olanzapine treatment for the last 1 year with no positive symptoms.

**Conclusions:**

What is the most appropriate treatment choice after NMS? There is no easy, single answer to this question. Although we were aware that there have been reported NMS cases with olanzapine use for this patient it was chosen since it is one of the second-generation antipsychotics that the patient did not use by examining the treatment history. This case shows that this syndrome may develop even after a long and stable neuroleptic treatment and the patient may continue to benefit from different antipsychotic treatments.

**Disclosure of Interest:**

None Declared

